# The evaluation of depressive, anxiety symptoms and sleep disturbance in college students post- pandemic era across UAE: a multicentric survey

**DOI:** 10.3389/fpsyt.2024.1489292

**Published:** 2024-11-27

**Authors:** Fathima Firoz, Ammar Ali Saleh Jaber

**Affiliations:** Department of Pharmacy Practice, Dubai Medical University, Dubai Pharmacy College for Girls, Al Mizhar, United Arab Emirates

**Keywords:** COVID-19 epidemic, mental health, sleep status, insomnia risk, depression

## Abstract

The COVID-19 epidemic has had a significant worldwide impact, requiring measures such as quarantine, social isolation, and the closing down of educational institutions, increasing concerns about student safety. This study evaluated the mental health and sleep status of 201 college students in the UAE after the epidemic. The sample consisted primarily of females (72.1%) and students aged 21-23 years (42.3%), with the majority residing in Dubai (62.7%). The findings revealed unsettling levels of insomnia risk and significant gender variations in emotional states, with females displaying higher degrees of distress. These findings underscore the negative consequences of the pandemic on student well-being and the importance of having access to mental health care, as well as calling for specialized treatments to address the individual needs of various student groups.

## 1 Introduction

COVID-19, a highly contagious virus first identified in Wuhan, China, in December 2019, spread to over 200 countries, leading the WHO to declare it a global pandemic on March 11, 2020 (1). To curb the spread, most countries temporarily closed educational institutions, impacting over 60% of the global student population. According to UNESCO, 138 countries closed schools, affecting more than 1.5 billion children and youth, while 11 countries enforced localized closures (4).

These measures disrupted businesses and education, leading to increased mental health issues such as anxiety, depression, stress, sleep difficulties, and substance abuse among university students (5). Even in non-pandemic times, students face mental health challenges from academic pressure and financial stress. The pandemic exacerbated these issues as students transitioned to online learning, limiting access to in-person mental health resources and support (6).

In a survey on the psychological status of undergraduate college students in the UAE during the COVID-19 pandemic, 36.7% expressed extremely severe anxiety. The study identified anxiety as the most common psychological issue, affecting 72% of the participants before the pandemic, the anxiety prevalence among Emirati undergraduates was estimated at 55%. This increase underscores the significant impact of the pandemic on students’ mental health (7).

Polese et al. discovered a statistically significant link between menstruation changes and stress during the pandemic in an observational study of female medical students. According to the study, 57.1% of participants with cycle abnormalities experienced symptoms of depression, and 58.1% reported sleep disturbances among students (8). These findings show the pandemic’s considerable influence on mental health, notably how stress can cause menstruation fluctuations, increase depressive symptoms, and disrupt sleep among female students.

Unrecognized anxiety and depression can jeopardize students’ mental health, leading to social and behavioral issues, poor academic performance, neglect of hygiene, inadequate self-care, and low self-esteem (9). Students with these disorders are nearly five times more likely than adults to experience negative outcomes, such as skipping classes or submitting incomplete assignments. They may also show signs of helplessness, pessimism, and social withdrawal (10).

Various factors, including sleep duration, timing, quality, and insomnia, have been examined to compare sleep habits before and during COVID-19. Although several studies have assessed the pandemic’s impact on psychological stress and sleep, further replication and expansion are needed (11).

Research on UAE university students post-pandemic is limited. Therefore, this study aims to investigate their mental health and sleep status, addressing a critical knowledge gap. Understanding these factors is essential for developing effective interventions and support systems to enhance student well-being in the aftermath of COVID-19.

## 2 Method

### 2.1 Study design and population

An online survey was conducted among college students in the United Arab Emirates by using Google Forms, an electronic survey administration software service offered by Google (12). The survey link was sent to the participants using various social media platforms including WhatsApp, Instagram, and Facebook. The data collection in this study was carried out using a structured self-administered questionnaire, where the students were asked for their consent before participating.

The sample size was estimated using the Raosoft calculator where a minimum of 196 participants was required for this study with a 95.0% confidence level and a margin of error of 0.7 (13).

The sample consisted of full-time college students currently enrolled in various academic disciplines across the UAE. The inclusion criteria included university and college students pursuing diploma, undergraduate, graduate, or PhD degrees, as well as college or university graduates from the academic years 2021 to 2022 who were willing to take part in the online survey. Students who were not currently enrolled, those residing outside the UAE, and those who did not provide informed consent were excluded (14). Out of the total surveys distributed, 210 students voluntarily responded, and only 201 of them were included in the study after applying the exclusion criteria.

### 2.2 Variables and measures

The survey consisted of fundamental demographic questions, which covered aspects like age, gender, nationality, the specific emirate of residence, as well as current educational level, academic field, and academic year. Furthermore, it had questions related to living circumstances, which included living arrangements (are you living alone; yes or no), levels of family conflict (always, never, rarely, sometimes, usually), and financial difficulties (not at all, moderately, a little, quite a bit, extremely). The survey also employed two validated mental health assessment tools to evaluate anxiety and depression symptoms, as well as sleep disturbances.

#### 2.2.1 Psychological scales

##### 2.2.1.1 Positive and negative affect schedule

Positive activation (PA) and negative activation (NA), two crucial components of emotional experience, are well-measured by PANAS. Positive emotions like joy and excitement are reflected by PA, whilst negative emotions like guilt and anxiety are reflected by NA (15). These characteristics are essential for comprehending people’s total emotional health, especially in challenging times like the COVID-19 pandemic. Since PANAS is well-known for its validity and reliability in clinical research, it is a perfect instrument for determining emotional states that can distinguish between depression and anxiety (15). This study’s short, 20-item scale makes assessment quick and easy. Respondents rate each characteristic from 1 (very slightly) to 5 (extremely) on a Likert-style scale (16).

#### 2.2.2 Sleep scales

##### 2.2.2.1 Ford insomnia response to stress test

The FIRST questionnaire was selected to assess susceptibility to sleep disturbances induced by stress. This scale is useful in the context of the pandemic, where heightened stress levels due to social isolation, academic challenges, and uncertainty about the future may contribute to sleep disruptions among university students. The FIRST has high reliability (test-retest reliability = 0.92), ensuring consistent measurement of sleep disturbance risk across various stressors commonly encountered throughout the day and evening. The overall score is between 9 and 36. High FIRST scores suggest a greater susceptibility to sleep disturbance (17). By utilizing FIRST, this study aims to identify students who are more vulnerable to sleep disturbances, providing insights into the specific stressors that may exacerbate these issues post-pandemic.

### 2.3 Statistical analysis

All data were statistically evaluated using the SPSS program. (IBM SPSS Statistics, Version 29, 2022). Before each analysis, all data was checked for violations of statistical assumptions. Cronbach’s alpha coefficients were calculated to indicate scale reliability.

For categorical data, descriptive statistics were calculated in terms of frequency and percentages, and for continuous variables, means and standard deviations (SD). The Chi-square test was used to find the association between demographic characteristics with the student’s depression, anxiety, and stress categories between the two time frames. Student’s t-tests and univariate ANOVA were used to compare the means of the depression scores, the anxiety scores, and the stress scores. Binary logistic regression analysis was used to model the relationship between the sleep scales of both the time frames with the demographic data. Linear logistic regression was used between the psychological scales of the two time frames and the demographic data. For all statistical studies, a p-value of less than 0.05 was utilized as a cut-off for the statistical significance level (7).

## 3 Results

### 3.1 Socio-demographic characteristics

The socio-demographic characteristics of the 201 participants who responded to the survey indicate that most of the students are females (n=145, 72.1%), with the majority falling in the age range of 21-23 years (n=85, 42.3%). A significant portion of the participants reside in the emirate of Dubai (n=126, 62.7%). By their educational background, most of them are pursuing an undergraduate or bachelor’s degree (n=158, 78.6%), primarily in the field of healthcare (n=74, 36.8%), and predominantly in their 3^rd^ academic year (n=54, 26.9%). Furthermore, a majority of participants do not live alone (n=176, 87.6%), report moderate financial difficulties (n=66, 32.8%), and rarely experience family conflicts (n=85, 42.3%). **Table 1** shows the complete socio-demographic characteristics of the participants.

**Table 1:** Complete demographic characteristics.

Characteristic	Number (%)
Gender	
Male	56 (27.9%)
Female	145 (72.1)
Age Group	
15-17	2 (1%)
18-20	72 (35.8%)
21-23	85 (42.3%)
24-26	27 (13.4)
27-29	7 (3.5%)
Above 30	8 (4%)
Emirate	
Abu Dhabi.	19 (9.5%)
Dubai	126 (62.7%)
Sharjah	42 (20.9%)
Ajman	11 (5.5%)
Fujairah	1 (0.5%)
Ras Al Khaimah	1 (0.5%)
Umm Al Quwain	1 (0.5%)
Educational Level	
Diploma	10 (5%)
Bachelor’s/Undergraduate	158 (78.6)
Master’s/Postgraduate	33 (16.4%)
Educational Field	
Aeronautics and Aviation Science	2 (1%)
Public Relation and Administration	2 (1%)
Fine arts and design	13 (6.5%)
Humanities and social sciences	8 (4%)
Media and Communication	4 (2%)
Information Technology	6 (3%)
Business and Commerce	72 (35.8%)
Healthcare	74 (36.8%)
Engineering	7 (3.5%)
Computer Science	13 (6.5%)
Academic Year	
1^st^ year	50 (24.8%)
2^nd^ year	38 (18.9%)
3^rd^ year	54 (26.9%)
4^th^ year	37 (18.4%)
5^th^ year	13 (6.5%)
6^th^ year	9 (4.5%)
Living alone	
Yes	25 (12.4%)
no	176 (87.6%)
Financial Difficulties	
Not at all	45 (22.4%)
Moderately	66 (32.8%)
A little	54 (26.9%)
Quite a bit	30 (14.9%)
Extremely	6 (3%)
Family Conflicts	
Never	35 (17.4%)
Rarely	51 (25.4%)
Sometimes	85 (42.3%)
Usually	25 (12.4%)
Always	5 (2.5%)

### 3.2 FORD insomnia test

The FORD insomnia test generated sleep vulnerability scores, which were then divided into high and low-risk groups using a cutoff score of 12 (17–19), set at the median value. In the current timeframe, a substantial 90.5% (n=182) of participants are identified as being at “High Risk” for insomnia. Notably, during the pandemic, a slightly higher proportion of 12.9% were categorized as “Low Risk”, although most of the students 87.1% (n=175) still fell into the “High Risk” category. The comparative results are presented in **Table 2**.

**Table 2:** Categorization of insomnia risk group.

Current Time-frame Insomnia Risk Group	
High Risk	182 (90.5%)
Low Risk	19 (9.5%)
Pandemic Time-Frame Insomnia Risk Group	
High risk	175 (87.1%)
Low risk	26 (12.9%)

Independent sample t-test, ANOVA test, and Pearson’s Chi-Square test which was used to compare the mean scores (± SD) of the insomnia risk groups for both the time frame and the demographic data are presented in **Table 3**.

**Table 3:** ANOVA, independent T-test, and Chi-square test for FORD insomnia.

UNIVARIATE ANOVA			
Current Timeframe			
Factor	F-Statistic	P-Value	Effect Size (Partial Eta Squared)
Age Group	1.123	0.349	Small (0.028)
Emirates	2.281	0.038	Small-To-Medium (0.066)
Educational Level	1.596	0.205	Small (0.016)
Educational Field	0.780	0.635	Small (0.035)
Academic Year	0.759	0.580	Small (0.019)
Family Conflicts	1.822	0.126	Small (0.036)
Financial Difficulties	1.179	0.321	Small (0.024)
Pandemic Timeframe			
Factor	F-Statistic	P-Value	Effect Size (Partial Eta Squared)
Age Group	0.631	0.676	Small (0.016)
Emirates	1.267	0.274	Small (0.038)
Educational Level	1.359	0.259	Small (0.014)
Educational Field	2.226	0.022	Moderate (0.095)
Academic Year	0.863	0.507	Small (0.022)
Family Conflicts	1.594	0.177	Moderate (0.032)
Financial Difficulties	0.543	0.704	Small (0.011)
CHI-SQUARE TEST			
Current Timeframe			
Factor		Pearson Chi-Square	P-Value
Gender		2.118	0.146
Age Group		5.627	0.344
Emirates		13.245	0.039
Educational Level		3.189	0.203
Educational Field		7.129	0.624
Academic Year		3.837	0.573
Do You Live Alone?		0.070	0.791
Do You Face Family Conflicts?		7.207	0.125
Do You Have Financial Difficulties?		4.724	0.317
Pandemic Timeframe			
Factor		Pearson Chi-Square	P-Value
Gender		1.670	0.196
Age Group		3.201	0.669
Emirates		7.581	0.270
Educational Level		2.721	0.256
Educational Field		19.080	0.025
Academic Year		4.350	0.500
Do You Live Alone?		1.265	0.261
Do You Face Family Conflicts?		6.333	0.176
Do You Have Financial Difficulties?		2.204	0.698
INDEPENDENT T-TEST			
Current Timeframe			
Gender			
Group	T (Equal Variances Assumed)	T (Equal Variances Not Assumed)	Significance (One-Sided)
Female	1.456	1.286	0.073
Male		80.220	0.101
Do You Live Alone?			
Group	T (Equal Variances Assumed)	T (Equal Variances Not Assumed)	Significance (One-Sided)
Yes	0.264	32.322	0.396
No			0.391
Pandemic Timeframe			
Gender			
Group	T (Equal Variances Assumed)	T (Equal Variances Not Assumed)	Significance (One-Sided)
Female	1.291	1.179	0.099
Male		84.600	0.121
Do You Live Alone?			
Group	t (Equal Variances Assumed)	t (Equal Variances Not Assumed)	Significance (One-Sided)
Yes	-0.555	-0.437	0.290
No		27.711	0.333

Student T-Test showed that no statistically significant difference was detected between the gender, living conditions, and insomnia risk groups for both time frames. The univariate ANOVA test conducted for the insomnia risk groups in the current time frame revealed that age (p-value = 0.349), level of education (p-value = 0.205), educational field (p-value = 0.635), academic year (p-value = 0.580), family conflicts (p-value = 0.126) and financial difficulties (p-value = 0.321) had no statistically significant impact on the group, with small effect sizes. However, the factor where the participants reside in the country exhibited a small-to-medium effect and was statistically significant (p-value = 0.038).

Whereas univariate ANOVA performed during the pandemic time frame has shown that age (p-value = 0.676), participants residence (p-value = 0.274), educational level (p-value = 0.259), academic year (p-value = 0.507), levels of family conflicts (p-value = 0.177) and financial situations (p-value = 0.704) does not impact statistically significant, each with small effect sizes.

However, participants’ educational field had a moderate effect and was statistically significant (p-value = 0.022).

Pearson’s Chi-square tests further supported these findings, indicating that the participant’s Emirate of residence significantly influenced the risk groups of the current time frame, while the educational field significantly affected the pandemic time frame. Finally, binary logistic regression indicated that the model did not provide strong predictive power, as it did not significantly outperform a constant-only model.

### 3.3 PANAS test

The PANAS test considers emotional experiences where on average, participants reported higher positive affect scores during a typical week (28.4 ± 8.3) than during the pandemic (25.8 ± 8.5). While the average negative affect scores were similar during the pandemic (25.8408 ± 9.1) and during the week (25.1 ± 9.1).

Following the linear regression analysis conducted for both time frames, involving positive and negative emotional states, the findings revealed that for a typical week, the overall regression model for positive emotions was not statistically significant (p = 0.942). Furthermore, none of the predictor variables exhibited individual significance, as all p-values exceeded 0.05. In contrast, when examining negative emotions during a typical week, the overall regression model displayed significance (p < 0.001). The independent variables “Gender” (p = 0.001) and “Financial difficulties” (p = 0.030) emerged as individually significant predictors, with an R-squared (R^2) of 0.188, indicating that these variables accounted for a moderate portion of the variance in negative feelings in a normal week. The linear regression analysis results are presented in **Table 4**.

**Table 4:** Linear regression analysis for PANAS.

Variable	R-squared (R^2)	Model Significance (p-value)	Individual Significance (p-values)
Positive_score_week	0.018	0.942	All p-values > 0.05
Negative_score_week	0.188	< 0.001	Gender (p = 0.001) Financial difficulties(p = 0.030)
Positive_score_pandemic	0.061	0.203	All p-values > 0.05
Negative_score_pandemic	0.116	0.004	Gender (p = 0.023) Financial difficulties (p = 0.002)

During the pandemic, the regression analysis for positive affect resulted in a low R-squared (R^2) of 0.061, suggesting that the independent variables contributed only marginally to the variance. Additionally, the overall regression model was not statistically significant (p = 0.203), and none of the predictor variables achieved individual significance, with all p-values exceeding 0.05.

However, in the regression analysis for negative feelings during the pandemic, an R-squared (R^2) of 0.116 indicated that the independent variables accounted for a moderate proportion of the variance. The overall regression model demonstrated significance (p = 0.004), and the independent variables “Gender” (p = 0.023) and “Financial difficulties” (p = 0.002) were identified as individually significant predictors.

## 4 Discussion

This study investigates the prevalence and determinants of insomnia risk and emotional experiences among college students in the United Arab Emirates (UAE) using an online survey distributed via social media platforms, examining both current and pandemic timeframes.

The findings revealed that the majority of participants (90.5%) were categorized as “High Risk” for insomnia in the post-pandemic era, with 87.1% during the pandemic. And a positive shift in emotional well-being among participants post-pandemic (28.4 ± 8.3), contrasting with the comparable negative affect scores between the pandemic (25.8408 ± 9.1) and the post-pandemic period (25.1 ± 9.1).

Various statistical tests were conducted to explore the relationships between demographic factors and insomnia risk, as well as emotional states.

### 4.1 Insomnia risk and demographic factors

One of the most striking findings of the study is the alarmingly high prevalence of insomnia risk among college students in the post-pandemic era. An overwhelming 90.5% of participants were identified as being at “High Risk” for insomnia, with an increase of 3.89% from the pandemic.

Contrary to expectations, the persistence of high insomnia risk levels indicates that the effects of the pandemic continue to effect the college student population.

The analysis showed that gender, living conditions, age, level of education, educational field, academic year, family conflicts, and financial difficulties had no statistically significant impact on insomnia risk in the current timeframe. However, the participants’ residence in the country exhibited a small-to-medium effect and was statistically significant. The variations in sleep patterns by the emirate of residence may be attributed to differences in access to resources that support sleep hygiene and mental well-being.

During the pandemic, similar results were observed, except for participants’ educational field, which had a moderate effect and was statistically significant. While the relationship between academic disciplines and sleep disturbances is a complex and multifaceted issue, the results suggest that it was indeed a significant factor influencing insomnia risk among college students. Students in different academic fields experience varying levels and types of stressors. For instance, healthcare students, who represented a substantial proportion of the study participants, often face demanding academic schedules, rigorous coursework, and clinical rotations. These factors can contribute to heightened stress levels and interfere with establishing and maintaining healthy sleep routines (20–25).

### 4.2 Emotional states and pandemic effects

The PANAS test assessed participants’ emotional experiences and encouragingly found that they reported higher positive affect scores during a typical week compared to during the pandemic. This shift towards more positive emotions is a significant and uplifting factor. The increase in positive feelings post-pandemic can be attributed to the remarkable resilience and adaptability demonstrated by college students, as well as the proactive initiatives undertaken by the UAE government to enhance the mental well-being of students (26–28). The pandemic brought about unprecedented challenges, including abrupt shifts to online learning, social isolation, and uncertainty about the future (1–5). Despite these challenges, many students have managed to adapt to the new circumstances and find ways to maintain or regain positive emotions. These combined efforts have contributed to the improved emotional well-being of students in the post-pandemic era.

Linear regression analyses were conducted to examine the relationship between emotional states and various predictors. During a typical week and the pandemic, the regression model for positive emotions was not statistically significant, with none of the predictor variables showing individual significance. However, for negative emotions, the regression model was significant, with “Gender” and “Financial difficulties” being significant predictors for both the current and pandemic time frames.

These results shed light on two critical factors that play a pivotal role in shaping the emotional well-being of college students, irrespective of the external circumstances. It indicates that gender significantly impacts negative feelings, with female students reporting higher levels of negative emotions compared to their male counterparts. This observation is in line with existing research that suggests women are more likely to experience negative affect and emotional distress (29–31).

In discussing the findings, it’s important to acknowledge several limitations of this study, including potential biases from self-reported data, reliance on online survey methods that may exclude certain demographics, the cross-sectional design limiting causal inference, and the relatively small sample size, which may restrict the generalizability of findings to broader student populations in the UAE.

## 5 Conclusion

The study sheds light on the mental health and sleep status of UAE university students in the aftermath of the COVID-19 pandemic. The study aimed to achieve several objectives, including evaluating depressive and anxiety symptoms and investigating the correlations between sleep disturbances and mental health issues.

The findings revealed a significant concern regarding insomnia risk, with a staggering 90.5% of participants categorized as being at “High Risk” for insomnia in the post-pandemic era, indicating a concerning 3.89% increase from the pandemic period.

On a more positive note, the study revealed a significant improvement in positive emotional states among college students post-pandemic. This increase in positive feelings can be attributed to the resilience and adaptability demonstrated by these students in the face of unprecedented challenges. The linear regression analyses indicated that gender and financial difficulties significantly impacted negative emotions in both the current and pandemic time frames, with female students reporting higher levels of negative emotions.

In summary, our research emphasizes the critical requirement for targeted mental health and sleep hygiene interventions to tackle the elevated risk of insomnia among college students in the UAE following the pandemic. It is of vital importance that mental health experts consistently monitor the emotional well-being of college students, particularly those at risk, and improve the accessibility of mental health services within the university (7, 12, 32). Relevant awareness should be created through various activities and social media platforms regarding healthy sleeping habits. These findings lay the groundwork for specialized interventions, support systems, and future research aimed at strengthening the overall welfare of the student demographics in the UAE.

## 6 Limitations

The use of self-reported data may induce biases, and the online survey approach may exclude specific demographics, limiting the generalizability of the findings. The cross-sectional methodology limits causal inference, and other external variables such as continued financial constraints and residual pandemic effects were not considered.

## Data Availability

The original contributions presented in the study are included in the article/supplementary material. Further inquiries can be directed to the corresponding author.
